# Biophysical and Biochemical Characterization of the Binding of the MarR-like Transcriptional Regulator Saro_0803 to the *nov1* Promotor and Its Inhibition by Resveratrol

**DOI:** 10.3390/biom13030541

**Published:** 2023-03-16

**Authors:** Zhen He, Zunhui Ke, Wei Wang, Yahui Liu, Haoran Zhang, Yan Li

**Affiliations:** 1 Department of Pathogen Biology, School of Basic Medicine, Tongji Medical College, Huazhong University of Science and Technology, Wuhan 430030, China; 2 Department of Blood Transfusion, Wuhan Children’s Hospital, Tongji Medical College, Huazhong University of Science & Technology, Wuhan 430016, China; 3 Medical Subcenter of HUST Analytical & Testing Center, Huazhong University of Science and Technology, Wuhan 430030, China; 4 Department of Pediatrics, Tongji Hospital, Tongji Medical College, Huazhong University of Science and Technology, Wuhan 430030, China

**Keywords:** multiple antibiotic resistance, transcriptional factor, repressor, NMR, resveratrol

## Abstract

Saro_0803 is a transcriptional factor modulating the transcription of the stilbene-degrading enzyme gene *nov1* in *Novosphingobium aromaticivorans* DSM 12444. Reportedly, Saro_0803 undergoes resveratrol-mediated dissociation from the *nov1* promotor and distinguishes resveratrol from its precursors, *p*-coumaric acid and trans-cinnamic acid, enabling the transcriptional factor to serve as a biosensor component for regulating resveratrol biosynthesis. However, little is known about the molecular mechanisms underlying the Saro_0803 interactions with either the *nov1* promotor gene or resveratrol, which undermines the potential for Saro_0803 to be further modified for improved biosynthetic performance and other applications. Here, we report the discovery of the 22 bp A/T-rich Saro_0803 binding site near the −10 box of the *nov1* promotor (named *nov1p*^22bp^). As validated by molecular docking-guided mutagenesis and binding affinity assays, the Saro_0803 binding of its target DNA sequence relies on charge-predominating interactions between several typical positively charged residues and nucleic acid. Furthermore, we semi-quantified the influence of resveratrol presence on Saro_0803–*nov1p*^22bp^ interaction and identified a bilateral hydrophobic pocket within Saro_0803 comprising four aromatic residues that are crucial to maintaining the resveratrol binding capability of the transcriptional factor. Our data are beneficial to understanding saro_0803′s structural and functional properties, and could provide theoretical clues for future adaptations of this transcriptional factor.

## 1. Introduction

The multiple antibiotic resistance regulator (MarR) family consists of an assortment of transcriptional factor (TF) proteins that are widely distributed among bacteria and act as repressors of genes that encode efflux pumps. MarR family TFs enable bacteria to adapt to environmental fluctuations, regulate the gene expression of diverse progress, and are involved in antibiotic resistance, oxidative stress agents, aromatic catabolism, organic solvents, and virulence [1,2,3,4,5]. The transcriptional factor Saro_0803, which was recently predicted to be a member of the MarR family, was initially identified as a component of the noncarotenogenic bacterium *Novosphingobium aromaticivorans* DSM 12444 and found to be encoded by the gene *saro_0803* that is located reversely on the upstream of gene *nov1* [6].

The chemical-signal-sensing function of MarR proteins relies on their capability of intergenic region binding and ligand-induced dissociation. MarR proteins exist in free or DNA-bound states, where a conserved winged helix mediates sequence-specific DNA binding. In most cases, this association can be attenuated by specific anionic lipophilic ligands [7,8,9,10]. In the case of Saro_0803, it was previously revealed that Saro_0803 binds to a cognate sequence overlapping the −35 and −10 boxes of the promoter of gene *nov1* encoding the stilbene-degrading enzyme NOV1. The NOV1 enzyme of *Novosphingobium aromaticivorans* DSM 12444 cleaves the interphenyl double bond of stilbenes with a 4′- oxygen functional group via monooxygenase or dioxygenase reactions, which is a supporting foundation for *Novosphingobium* to degrade various aromatic compounds [11,12,13]. In the presence of resveratrol, a stilbenoid substrate of NOV1, the binding of Saro_0803 to *nov1* promotor can be weakened to activate the downstream *nov1* transcription, suggesting the involvement of Saro_0803 in the feedback regulation of NOV1 [12,14,15]. Interestingly, as one of the most well-characterized stilbenoids, resveratrol is naturally produced by several plant species, which is mainly attributed to its potential binding to a rich diversity of molecular targets involved in anti-oxidative, anti-inflammatory, and anti-cancer activities [16,17,18,19,20,21].

As a transcriptional factor, Saro_0803 has been reported to serve as a gating biosensor for stilbene/cannabinoid biosynthesis [6]. However, the molecular mechanism underlying both the binding of Saro_0803 to the *nov1* promoter and its attenuation by resveratrol is poorly understood, posing an obstacle to the further development of Saro_0803-related biosensors. In this study, by biochemical, biophysical, and simulation characterizations, we present the molecular properties of Saro_0803 that allow it to specifically bind the core sequential fragment *nov1p*^22bp^ of *nov1* and propose that Saro_0803 is likely to accommodate resveratrol as a ligand with an aromatic pocket, and thus, loses the tight binding of the target DNA fragment *nov1p*^22bp^. Our research is expected to facilitate the mechanical understanding of the *nov1*-Saro_0803–resveratrol regulation system and to provide a necessary theoretical basis for further modifications and applications of Saro_0803.

## 2. Materials and Methods

### 2.1. Plasmid Constructs

The gene encoding *saro_0803* was cloned and inserted into the vector pET-30a(+) using the Nde I/Xho I restriction sites. All mutants were constructed by site-directed mutagenesis using PCR primers listed in Table S1. Recombinant plasmids were transformed into *E. coli* BL21(DE3) strain for protein expression.

### 2.2. Protein Expression and Purification

All Saro_0803 proteins, including the wild type and mutants, were cultured in LB medium with 50 µg/mL kanamycin at 37 °C at 200 rpm until absorbance at 600 nm (OD_600_), reaching ~0.6–0.8. Overexpression was conducted at 18 °C at 200 rpm for 16 h by induction with 0.2 mM isopropylthiogalactoside. Cells were then collected and lysed by a vacuum disruptor in binding buffer (50 mM Tris-HCl pH 8.0, 500 mM NaCl, 10 mM imidazole, 1 mM PMSF), then the lysate was centrifuged at 18,000× *g* at 4 °C for 30 min. The protein was purified via Ni-affinity chromatography by loading the supernatant onto a Ni-NTA column (fast flow, Qiagen) eluted with the elution buffer (50 mM Tris-HCl pH 8.0, 500 mM NaCl, and 300 mM imidazole). Subsequently, protein purification was performed by size exclusion chromatography on a Superdex 200 increase 10/300 GL column (GE Healthcare, Chicago, IL, USA) using the buffer (50 mM Tris-HCl pH 8.0, 200 mM NaCl), and then the fractions were collected and concentrated. Then, the collected fractions were determined by Tricine SDS-PAGE, and protein concentrations were measured with a NanoDrop2000 spectrophotometer (Thermo Scientific, Waltham, MS, USA).

### 2.3. Analytical Ultracentrifugation (AUC) Experiments

The sedimentation velocity (SV) experiment was carried out in an ProteomeLab XL-I AUC instrument (Beckman Coulter Life Sciences, Indianapolis, IN, USA). The protein sample (~1.0 mg/mL) was loaded into an ultracentrifuge tube, then centrifuged at 40,000 rpm for 2 h at 4 °C, unloaded immediately after centrifugation, resulting in a continuous gradient. Data were collected in intensity mode, and the sedimentation coefficients and the continuous c(s) distribution were analyzed using the Sedfit software [22].

### 2.4. Nuclear Magnetic Resonance (NMR) Experiments

The vector pET-30a (+)-*saro_0803* was transformed into *E. coli* BL21(DE3) strain for protein expression. For the production of uniformly [^15^N]-labeled Saro_0803, the protein is expressed and purified as described in Section 2.2, except that the cells were grown in a minimal medium containing 1 g/L ^15^NH_4_Cl for [^15^N]-labeling, and prepared in an NMR buffer containing 20 mM MES pH 6.5, and 200 or 600 mM NaCl, 5% (*v*/*v*) D_2_O. One-dimentional [^1^H] and two-dimensional [^1^H-^15^N]-HSQC NMR spectra were acquired with Bruker Avance III spectrometers equipped with cryoprobes, with the proton resonance frequencies of 600 MHz, 700 MHz, and 850 MHz at 298 K. The pulse programs within the standard pulse library (Bruker, Karlsruhe, Germany) were used for NMR data collection. The NMR data were processed with Bruker Topspin 4.1.3 and NMRPipe [23], visualized and analyzed using Cara.

### 2.5. NMR Titration Experiments

To verify the interaction between Saro_0803 and the core DNA sequence *nov1p*^22bp^ as well as the interaction between Saro_0803^21-157C39S^ and the small ligand resveratrol, 2D [^1^H-^15^N]-HSQC NMR spectra were obtained using a Bruker Avance III 600 MHz spectrometer at 298 K. The protein Saro_0803 samples were prepared in an NMR buffer containing 20 mM MES pH 6.5, 200 mM NaCl, and 5% (*v*/*v*) D_2_O. The Saro_0803 sample was then temporarily transferred from the NMR tube into the sample tube containing dry DNA powder for equimolar titration. The protein sample of Saro_0803^21-157C39S^ was prepared in an NMR buffer containing 20 mM MES pH 6.5, 600 mM NaCl, and 5% (*v*/*v*) D_2_O, and we temporarily transferred the sample from the NMR tube into the sample tube containing resveratrol (CAS No. 501-36-0) for titration. Seven 2D [^1^H-^15^N]-HSQC spectra with protein: resveratrol ratios of 1:0, 1:0.5, 1:1, 1:2, 1:4, 1:8, and 1:16 were recorded to observe the composite chemical shift perturbations ΔH-N upon ligand binding [24].

### 2.6. Oligonucleotides Annealing

Single-stranded DNA oligonucleotides were purchased from Tsingke biological technology Co., Ltd. (Beijing, China) for in vitro assays. Double-stranded DNAs were prepared by annealing complementary oligonucleotides in buffer (50 mM Tris-HCl pH 8.0, 200 mM NaCl) to 95 °C and slowly cooling them to room temperature. Detailed sequences are listed in Tables S2–S4.

### 2.7. Electrophoretic Mobility Shift Assays (EMSA)

DNA binding was assayed using Electrophoretic Mobility Shift Assays (EMSA) with a constant 5.0 µM DNA concentration and increased protein concentrations. Saro_0803 and the mutants were incubated with DNA at 37 °C for 30 min to allow complex formation. Subsequently, complexes were loaded onto 4–20% Mini-PROTEAN^®^ TGX™ Precast Gel (Bio-Rad, Hercules, CA, USA) and separated in Tris/Glycine buffer using 100 V/50 min on ice. The gel was soaked in TAE buffer with SYBR^®^ Safe stain (Invitrogen™, Eugene, OR, USA), shaken for 15 min for staining, and photographed using the Bio-Rad Molecular Imager^®^ Gel Doc^TM^ XRS system (Bio-Rad, Hercules, CA, USA).

### 2.8. Isothermal Titration Calorimetry (ITC) Assay

Experiments with expressed Saro_0803 and related mutants were performed using a MicroCal iTC200 instrument (Malvern Instruments Ltd., Malvern, UK)**.** The titration buffer was made of 50 mM Tris-HCl pH 8.0, 200 mM NaCl; 5% DMSO), with 20 µM protein in the cell, and 200 µM DNA or 2 mM resveratrol in the syringe at 25 °C. Seventeen repeats of 2.2-µL injections were performed at intervals of 120 s. The titration data were analyzed with the software Origin 9.0 and fitted using a one-site binding model.

### 2.9. Analytic Size Exclusive Chromatography (SEC)

Analytical size exclusion chromatography (SEC) was performed utilizing Superdex 200 increase 10/300 GL column (GE Healthcare, Chicago, IL, USA) equilibrated with a buffer (50 mM Tris pH 8.0, and 200 mM NaCl). Saro_0803, truncated Saro_0803^21-157^, and DNA *nov1p*^22bp^ samples at respective 100 µM concentrations were analyzed via SEC as the control. Experimental groups include co-incubated 200 µL protein-DNA samples at 100 µM + 100 µM and 100 µM + 150 µM, respectively. The elution was collected for analysis.

### 2.10. Molecular Docking

Before performing the molecular docking of Saro_0803 with *nov1p*^22bp^, the predicted structure of Saro_0803 was retrieved from *AlphaFold2* (https://alphafold.ebi.ac.uk/, accessed on 21 February 2022) [25], and the double-stranded *nov1p*^22bp^ fragment was generated via Avogadro 1.2.0 [26]. Then, the interaction models were created by the HDOCK server (http://hdock.phys.hust.edu.cn/, accessed on 22 February 2022) [27,28]. The HDOCK server performed protein–DNA docking with a hybrid docking algorithm of template-based modeling and free docking. Predicted Saro_0803–*nov1p*^22bp^ complexes were ranked by the iterative knowledge-based scoring function ITScore-PP. The complexes with lower energy scores were selected and visualized using PyMOL^TM^ 2.5.4 (Schrödinger, Inc., New York, USA).

### 2.11. Surface Plasmon Resonance (SPR)

The binding kinetics of purified His-tagged Saro_0803 to the resveratrol was measured by surface plasmon resonance (Open SPR^TM^, Nicoyalife, Kitchener, Canada). Once the NTA sensor chip was activated, His-tagged Saro_0803 was captured with the sensor chip to achieve a stable baseline. Diluted resveratrol with different concentrations was loaded to flow slowly over the sensor chip for 240 s, allowing protein–ligand interactions to occur. Lastly, the running buffer was allowed to flow for 360 s to collect the dissociation data. The flow rate of all solutions over the sensor chip was set to 20 µL/min at 25 °C. The binding kinetic parameters were obtained by fitting the signal response vs. time curve to a one-to-one binding model using TraceDrawer (Ridgeview Instruments AB, Uppsala, Sweden) software.

## 3. Results

### 3.1. Biochemical and Biophysical Characterization of Saro_0803

**Figure 1:**
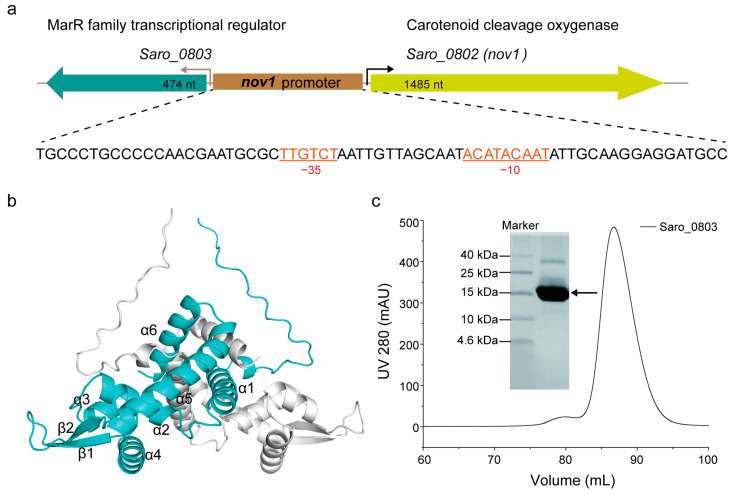
Biochemical and biophysical characterization of Saro_0803. (**a**) The gene organization of the resveratrol catabolic operon. The single oligonucleotide sequence of the *nov1* promoter is displayed underneath, with canonical −10 and −35 boxes indicated (colored in orange). (**b**) The monomeric units in the structural model of Saro_0803 predicted by *AlphaFold2* are colored in cyan and gray, respectively. (**c**) The expression and purification of transcriptional regulator Saro_0803 *in vitro*. Saro_0803 was purified by size exclusion chromatography using the HiLoad 16/600 Superdex 200 pg column and analyzed by tricine-SDS-polyacrylamide gel.

The gene *saro_0803* encodes the transcriptional regulator Saro_0803, and conversely, is located upstream of *nov1* (*saro_0802*), between which the *nov1* promoter possesses canonical −10 and −35 boxes [6] (Figure 1a). The multiple sequence alignment indicated that Saro_0803 shows sequence similarity to *E. coli* MarR of up to 37.0%, and the relative conservation of secondary structural elements of MarR families (Figure S1) [6,29].A protein structure prediction AI system *AlphaFold2* was utilized to predict the structure of Saro_0803. In this structure, Saro_0803 is characterized as triangular in shape with a pseudo-two-fold axis of symmetric assembly, a structural feature shared with MarR family member proteins. This model suggests a dimerization pattern, where helices α1, α5, and α6 of both monomeric units pack into a compact helical bundle, which is comparable to those of other MarR members [30,31] (Figure 1b and  Figure S2).

To further investigate the structural and functional features of Saro_0803, we successfully expressed and purified the polyhistidine-tagged Saro_0803. SDS-PAGE showed that the purified protein has an experimental molecular weight (M_W_) slightly larger than 15 kDa, consistent with its theoretical M_W_ of around 18.75 kDa (Figure 1c). The size-exclusion chromatography results indicate that the protein was generally homogeneous in the solution. For the detailed illustration of the oligomerization state, analytical ultracentrifugation (AUC) was then utilized to detect the sedimentation and the aggregation of Saro_0803, by which Saro_0803 was found to form a homogeneous dimer (Figure S3), supporting our *AlphaFold2*-based structural prediction result. Next, we uniformly purified ^15^N-labeled Saro_0803 samples for the heteronuclear-NMR-based depiction of structural characteristics. The 1D [^1^H] spectrum and the 2D [^1^H-^15^N]-HSQC spectrum of Saro_0803 showed dispersed peaks (Figures S4 and S5, respectively), suggesting that Saro_0803 was well folded in the solution and, therefore, suitable for further biochemical and biophysical studies. Nevertheless, the limited number of resolvable amide peaks and the inhomogeneity of intensity among the peaks in the [^1^H-^15^N]-HSQC spectrum also hint at a complicated, if not dimeric, state of Saro_0803 in the solution (Figure S6).

### 3.2. Identification of a 22 bp Core Binding Sequence of nov1 Promoter with Saro_0803

**Figure 2:**
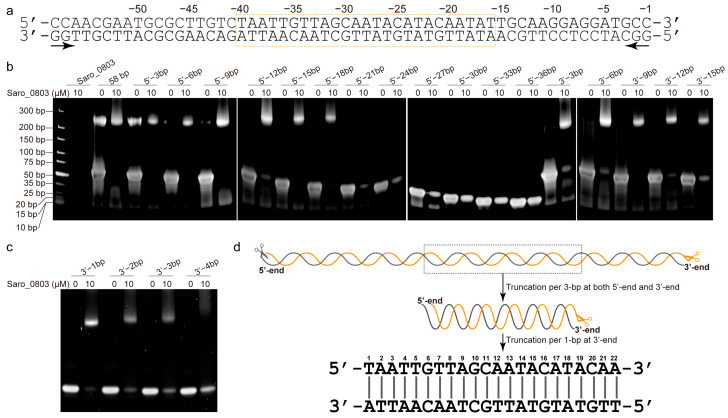
Identification of a 22 bp core binding sequence of *nov1* promoter with Saro_0803. (**a**) The double-stranded oligonucleotide sequence with a 58 bp truncation of the *nov1* promoter. The bilateral arrow below the sequence indicates the two opposite directions of the serial 3 bp truncations. The box in orange indicates the 25 bp middle section (pos. from −16 to −34) of the *nov1* promoter, which is speculated to be the truncation with a critical length that retains Saro_0803 binding ability. (**b**) EMSA analysis of the binding site of Saro_0803. The concentration of Saro_0803 with 0 µM or 10 µM incubated with 5.0 µM truncated dsDNA fragments of *nov1* promoter with serial lengths. (**c**) EMSA analysis indicating the binding site of Saro_0803 on *nov1* promoter by deletion from 3′-end of the 25 bp middle section. The concentration of Saro_0803 is labeled in the same manner as in (**b**). (**d**) The illustration of the core sequence of *nov1p*^22bp^ revealed by the repeated truncation performed on the original 58 bp truncation of the *nov1* promoter.

The *nov1* promoter (*nov1p*) possesses a length of 68 bp and includes an inverted repeat, GCAATA------TATTGC, overlapping with the predicted −10 box (Figure 1a) [6]. To track down the accurate location of the binding site of Saro_0803 and the minimal sequence of the *nov1* promoter for effective binding, essentially, a series of truncated, double-stranded DNA (dsDNA) sequential fragments were designed and investigated for the interaction of the purified Saro_0803 and the 58 bp *nov1* promoter fragment by an electrophoretic mobility shift assay (EMSA) (Figure 2a, Tables S2–S4). Roughly, we designed the fragments by progressive truncation at both the upstream and the downstream of the 58 bp dsDNA, truncating a 3 bp length at each step. Remarkably, with the 21 bp deletion at the 5′-end, the remaining 37 bp fragment exhibited a completely disappeared Saro_0803 binding ability. Similarly, the 15 bp deletion at the 3′ -end showed a neutralized Saro_0803 binding ability (Figure 2b). Hence, the specific Saro_0803 binding site spans a 25 bp middle section of the *nov1* promoter (Figure 2a). Then, we designed the 25 bp middle section dsDNA and performed 1 bp progressive truncation to it at the 3′-end. The assays indicated that the deletion by the length of 4 bp resulted in the loss of Saro_0803 binding (Figure 2c,d). These observations suggested that the binding of Saro_0803 to the core sequence with an approximate length of 22 bp is located downstream of the −35 box and occupies the −10 box (hereafter referred to as *nov1p*^22bp^). Further, we tested two neighboring DNA fragments against the *nov1p*^22bp^ sequence via EMSA to feature their Saro_0803 binding abilities (Figure S7), where *nov1p*^22bp^ was the only one out of three fragments that formed a complex with Saro_0803, proving the binding-site specificity of the TF. Additionally, the analytic size exclusion chromatography experiments confirmed that full-length Saro_0803 and its truncation Saro_0803^21-157^ bound to the dsDNA sequence *nov1p*^22bp^ (Figure S8).

### 3.3. Characterization of the Binding Capability of Saro_0803 with nov1p^22bp^

**Figure 3:**
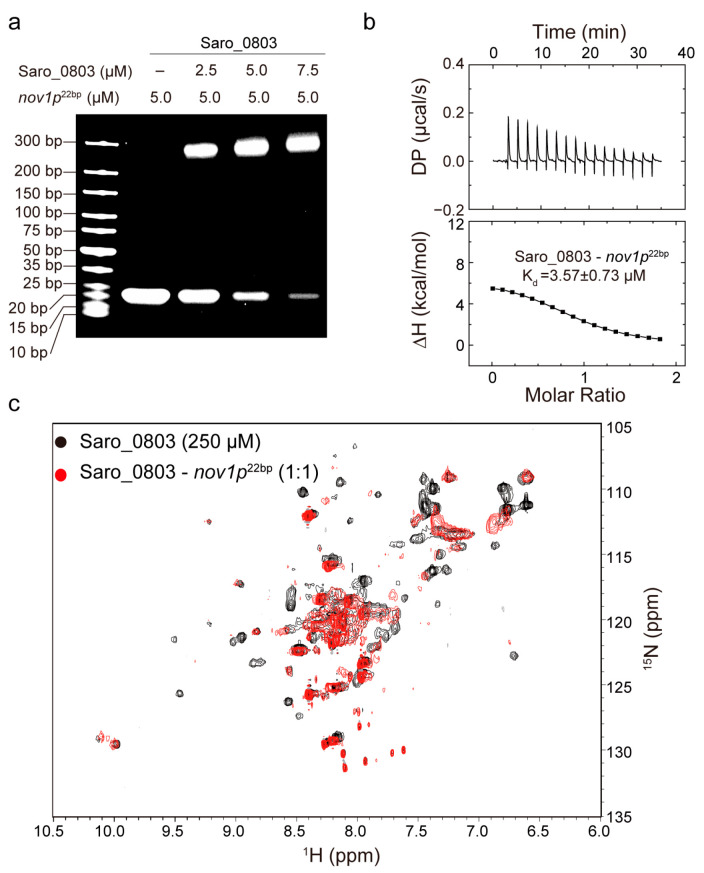
Characterization of the binding capability of Saro_0803 with *nov1p*^22bp^. (**a**) EMSA analysis of the Saro_0803–*nov1p*^22bp^ binding ability. Incrementally increased concentrations of Saro_0803 (0, 2.5, 5.0, and 7.5 µM) were incubated with 5.0 µM *nov1p*^22bp^. (**b**) ITC assay with continuous titration of *nov1p*^22bp^. (**c**) NMR titration experiment. Superimposed 2D [^1^H-^15^N]-HSQC spectra of Saro_0803 in the absence (black) and presence of incubated *nov1p*^22bp^ (red).

To clarify the Saro_0803 binding capability to the core sequence of the *nov1* promoter, we first carried out EMSA experiments between *nov1p*^22bp^ and increased concentrations of Saro_0803. Additionally, the semi-quantitative results showed that 5.0 μM Saro_0803 binds to more than half of the *nov1p*^22bp^ at an equimolar ratio of *nov1p*^22bp^ (Figure 3a). The detailed characterization of Saro_0803–*nov1p*^22bp^ binding affinity was later explored by ITC analysis and other biophysical methods. Using ITC assays, we found that Saro_0803 binds to the core sequence with a micromolar affinity (K_d_ ~3.57 µM) (Figure 3b). In addition, NMR experiments were utilized to explore the binding of the Saro_0803 and *nov1p*^22bp^. We acquired [^1^H-^15^N]-HSQC spectra of Saro_0803 with and without dsDNA *nov1p*^22bp^ co-incubation for comparative verification. A major reduction of the intensity was observed for many amides group cross peaks in the spectrum, indicating an intermediate chemical exchange, typically with a micromolar interaction of Saro_0803 induced by *nov1p*^22bp^ (Figure 3c). Although we could not achieve the full backbone assignments of Saro_0803, the observation that only a limited number of cross peaks obviously indicates that *nov1p*^22bp^ has a specific interaction with Saro_0803, which is consistent with other MarR family members. In conclusion, different biochemical and biophysical results suggested that Saro_0803 binds to *nov1p*^22bp^ with a micromolar affinity.

### 3.4. Molecular Docking-Based Investigation of the Molecular Mechanism Underlying Binding of Saro_0803–nov1p^22bp^

**Figure 4:**
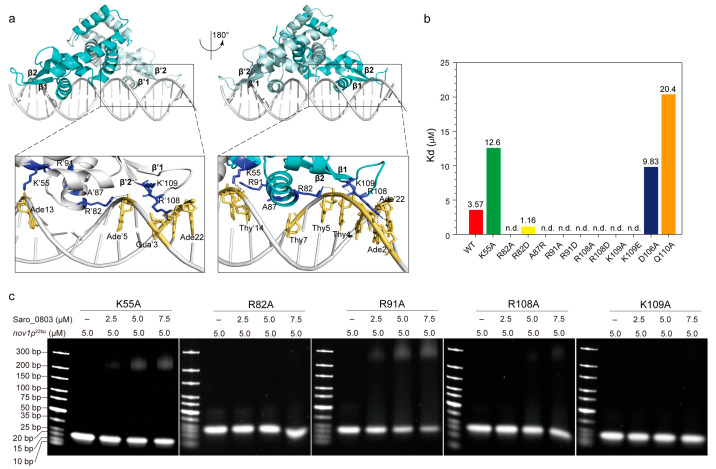
Investigation of the interaction pattern of Saro_0803 binding with *nov1p*^22bp^. (**a**) Overall, the molecular docking model of Saro_0803 bound to its operator DNA sequence *nov1p*^22bp^, represented in two orientations with 180° rotation. The dark box indicates the location of protein–DNA interactions. Close-up view of the bilateral protein–DNA interactions, where residue K55 and the residues of helix α4/α′4 are bound to the major groove and a looped region between strands β1-2/β′1-2 to the minor groove, respectively. (**b**) The colored column diagram of binding affinity (K_d_) of the interaction between the wild type or mutants of Saro_0803 and *nov1p*^22bp^ determined by ITC experiments. (**c**) EMSA analysis of the interaction between the wild type or mutants of Saro_0803 and *nov1p*^22bp^, where the increased concentration of protein with 0, 2.5, 5.0, and 7.5 µM were incubated with 5.0 µM DNA sequence *nov1p*^22bp^.

We next sought to explore the molecular mechanism of Saro_0803 binding to the core sequence of the *nov1* promoter. As the molecular docking approach is widely applied for studying biologically relevant molecular interactions, we conducted free docking between Saro_0803 and *nov1p*^22bp^ via HDOCK to characterize their interaction pattern [28]. Subsequently, a docking model with rough rotational symmetry was generated with our information, where the spatial interactions could be concluded as major groove-oriented and minor groove-oriented contacts. Residues such as R82 and R91 in helices α4/α′4 and K55 in helices α2/α′2 of Saro_0803 are shown to have contacts with the major groove of DNA *nov1p*^22bp^, while residues such as R108 and K109 located in the interloop between β1 and β2 are distributed near the minor groove and disposed to the grooves at both sides of *nov1p*^22bp^, which is positively charged in the residues and induce specific interactions with its cognate DNA sequence (Figure 4a). This docking model exhibits a binding pattern predominated by charge contacts between the positively charged residues of Saro_0803 and the negative-charge-enriched *nov1p*^22bp^ fragment. In order to validate the reliability of this model, we performed an in-depth analysis of the binding pattern, followed by site-directed mutation combined EMSA and ITC assays for verification.

The insertion of helices α4/α′4 to the DNA major grooves was a key part of the binding pattern that was worthy of further investigation. K55, R82, and R91, the contribution to α4/α′4-major groove interactions, clamped the DNA fragment by targeting its backbone phosphate groups, potentially stabilizing the overall protein–DNA interaction (Figure 4a). Since the charge-related interactions play an important role in the docking model, we designed mutations for lysine and arginine into both the negatively charged residues and alanine. Accordingly, we found that the mutation of K55 to an alanine led to a three-fold decrease in the Saro_0803–*nov1p*^22bp^ binding affinity, while the R82A/R82D and R91A/R91D mutants, measured by EMSA and ITC, showed a dramatically decreased binding affinity (Figure 4b,c, Figures S9 and S10). These results are supportive of our docking model, suggesting that the loss of positively charged guanidyls deforms the anchorage of Saro_0803 to the corresponding negative charges of *nov1p*^22bp^. On the contrary, we introduced the mutant A87R, as A87 is a neutral residue in α4 that is spatially close to the negatively charged regions of *nov1p*^22bp^. Interestingly, A87R was found to be a binding-enhancing mutation (K_d_ ~1.16 µM), further strengthening the credibility of our docking model (Figure 4b, Figures S9 and S10).

The inspection of the loop region between strands β1–β2 led to the focus on R108 and K109, two positively charged residues inserted into the minor groove near the *nov1p*^22bp^ termini. By examining our docking model, we noticed R108 guanidyl might be engaged in hydrogen bonds with the pyrimidine rings of thymine Thy′20 and Thy′21 and the 4-hydroxyl of deoxyribose of Ade′22, while K109 is close to its nearby phosphate groups of the minor groove backbone. Moreover, later affinity tests revealed that the binding constants of both R108 mutants (R108A and R108D) and K109 mutants (K109A and K109E) were not detectable (Figure 4a–c, Figures S9 and S10). These results agree with the model where the loss of positively charged side chains of R108 and K109 might weaken minor groove binding (Figure 4a). Additionally, D106 and Q110, which are located close to the R108–K109 pair, but were not observed to engage in direct contact with the minor grooves, were also chosen for mutagenesis. The mutants D106A and Q110A were reported to lower the binding affinity, indicating their role as auxiliary residues for Saro_0803–*nov1p*^22bp^ binding or as secondary binders that might rely on certain transient conformations to interact with the DNA minor grooves (Figure 4b, Figures S9 and S10). In summary, the EMSA and ITC binding assays for the above Saro_0803 mutants provided experimental support for our docking model and laid an essential basis for the explicit characterization of molecular mechanisms for Saro_0803–*nov1p*^22bp^ binding.

### 3.5. Exploration of the Mechanism of Resveratrol Binding to Saro_0803

A previous study revealed that the presence of resveratrol weakens the binding of Saro_0803 to a cognate sequence on the *nov1* promoter, and Saro_0803 can respond to altered resveratrol production within the cell and take the role in stilbene-responsive regulation of the expression of the stilbene-degrading enzyme NOV1 [6]. However, the molecular mechanism of resveratrol interference of Saro_0803 regulating the *nov1* promoter has not been revealed so far.

**Figure 5:**
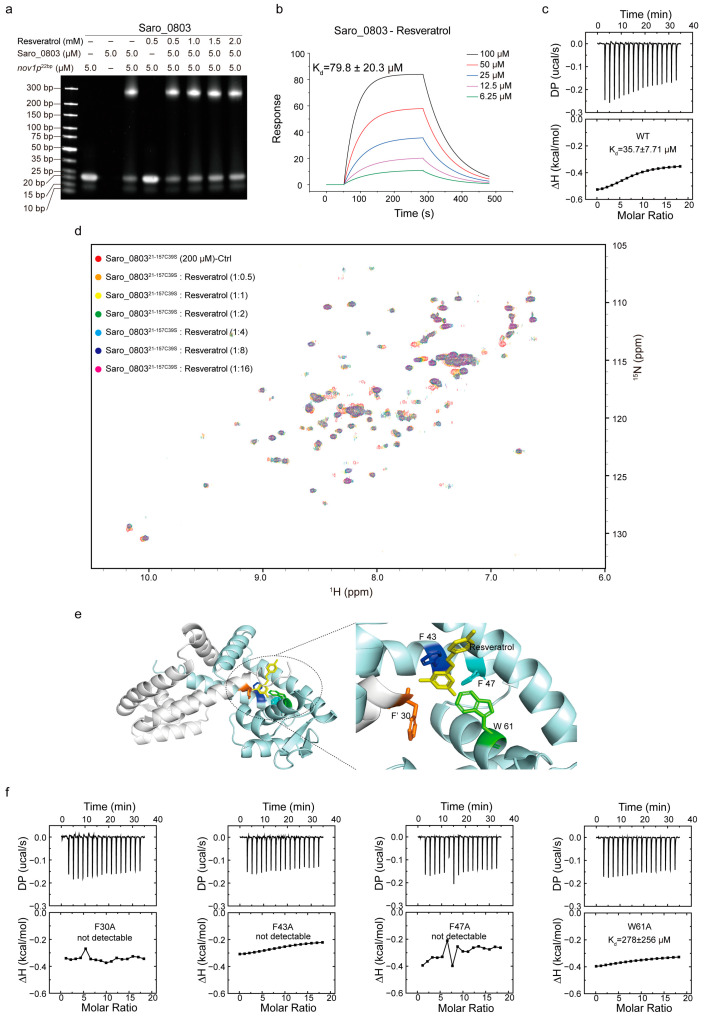
Exploration of the mechanism of resveratrol binding to Saro_0803. (**a**) EMSA analysis to investigate the ligand resveratrol perturbing the Saro_0803–*nov1p*^22bp^ binding ability. Incrementally increased concentrations of resveratrol (0, 0.5, 1.0, 1.5, and 2.0 mM) were incubated with 5.0 µM Saro_0803–*nov1p*^22bp^ complex. (**b**) SPR experiment with continuous titration of increased concentrations of resveratrol. (**c**) ITC assay of the Saro_0803 titrated with increased concentrations of resveratrol. (**d**) NMR titration experiment illustrated by superimposed 2D [^1^H-^15^N]-HSQC spectra of Saro_0803^21-157C39S^ in the absence (red) and presence of incubated resveratrol with increased concentrations, where the protein–ligands ratios were at 1:0.5, 1:1, 1:2, 1:4, 1:8, and 1:16 orange, yellow, green, cyan, blue, and magenta, respectively. (**e**) Molecular docking model of Saro_0803 bound to resveratrol (left panel), and local insight focusing on the presumed protein–ligand binding hotspot (right panel), with the Saro_0803 residues F30, F′43, F′47, and W′61 and resveratrol colored in orange, blue, cyan, green, and yellow, respectively. (**f**) ITC assays of the mutants of Saro_0803 titrated with increased concentrations of resveratrol.

With the characterization of Saro_0803–*nov1p*^22bp^ binding, we advanced the exploration of the Saro_0803–resveratrol binding feature and how the ligand binding could attenuate the specific DNA binding of Saro_0803. Initially, we performed the EMSA analysis and revealed that resveratrol could inhibit the binding between Saro_0803 and the core sequence *nov1p*^22bp^. Despite the change in the band brightness representing Saro_0803 binding to *nov1p*^22bp^ not being clear between increased resveratrol concentrations, non-Saro_0803-binding *nov1p*^22bp^ bands were observed to display enhanced brightness under higher resveratrol concentrations (Figure 5a). These modest changes in the gel bands might be attributed to the limited effective resveratrol concentration due to insufficient solubility within the buffer, in which the DMSO concentration was restricted to a maximum of 5% (*v*/*v*) to keep Saro_0803 in a compatible environment. We then found the binding affinity of Saro_0803 to resveratrol to be K_d_ ~79.8 µM by SPR and ~35.7 µM by ITC, indicating micromolar binding (Figure 5b,c). Furthermore, we conducted NMR titration experiments and found that certain amide group cross peaks of Saro_0803^21-157C39S^ in [^1^H-^15^N]-HSQC underwent chemical shift perturbation and peak broadening after increased concentrations of resveratrol were added, practically denoting intermediate binding (Figure 5d).

Resveratrol (or 3,5,4′-trihydroxystilbene) is a stilbenoid and consists of two phenol-hydroxyl-bearing benzene groups linked by a vinylene group [17,32,33]. Compared to the abundantly negatively charged *nov1p*^22bp^, net charge distribution is rare within a resveratrol molecule, which might imply a different pattern for Saro_0803–resveratrol binding from the charge-predominating one for Saro_0803–*nov1p*^22bp^ binding. As the backbone of the resveratrol structure is speculated to be hydrophobic and pro-aromatic, we noticed that an aromatic pocket comprising residues F30 in α1, F′43, and F′47 in α′1, and W′61 in α′2 is a potential target within Saro_0803 for resveratrol binding due to stabilization from the possible π–π stacking effect, with spatially neighboring hydrophilic residues being likely to form hydrogen bonds with the phenol hydroxyl groups of resveratrol. Consequently, we employed molecular docking to provide insights into the Saro_0803–resveratrol interaction. The docking result shows that the lateral residue F30 and the contralateral F′43, F′47, and W′61 jointly form a sizeable internal cavity, partially accommodating resveratrol (Figure 5e).

We next sought to validate the above binding properties experimentally and, accordingly, applied alanine mutation to the residues F30, F43, F47, and W61 before ITC and EMSA measurements for mutant–resveratrol binding. It was found that compared to the wild-type Saro_0803, the binding affinity for the four mutants to resveratrol significantly decreased or reduced to an undetectable level (Figure 5f and Figure S11). Further, we discovered that these four mutants retained micromolar or submicromolar binding affinities to *nov1p*^22bp^ (Figure S12). Taken together, these data indicated the key role in resveratrol binding played by aromatic residues F30, F43, F47, and W61. Of note, due to the lack of protein backbone flexibility during the docking stage, the docking result might not have exhibited dynamic binding properties, such as the potential allosteric effect of Saro_0803 upon resveratrol binding. Summarily, our results shed light on further mechanistic studies on resveratrol-induced Saro_0803 dissociation from the *nov1* promotor in the future.

## 4. Discussion

Saro_0803, a putative member of the MarR family transcriptional factors, displayed distinctive potential as a valuable tool in synthetic biology. Reported as a gating sensor, Saro_0803 was incorporated into biosynthetic cell units to detect the concentration fluctuation of stilbenoids such as resveratrol and regulate their synthesis via a negative-feedback route [6]. However, explicit molecular mechanisms underlying the feedback regulation, especially the resveratrol-induced Saro_0803 dissociation from the TF’s binding motif upstream of the target gene, are not well established yet. Elucidation of these key mechanisms is expected to help modify the feedback system, achieving improved synthetic efficiency and substrate identification specificity.

In this study, we described a model where the MarR-like TF Saro_0803 binds a 22 bp cognate sequence *nov1p*^22bp^, which spans the sequence from −35 to −10 boxes of the *nov1* promoter, with sequential specificity and affinity at the micromolar level. This result might aid the future explanation of the binding mode variety and the niche-dependent functional discrepancies among the MarR family TFs. In our docking model of a Saro_0803–*nov1p*^22bp^ complex, the charge interactions between arginines/lysines and DNA backbone phosphate groups played a significant part in the binding interface. Differently, residues R108/R′108 were observed to stick to the nucleoside part of the minor groove. Nevertheless, to validate this speculation, the details of the binding dynamics of the Saro_0803–*nov1p*^22bp^ complex have been under intensive investigation.

**Figure 6:**
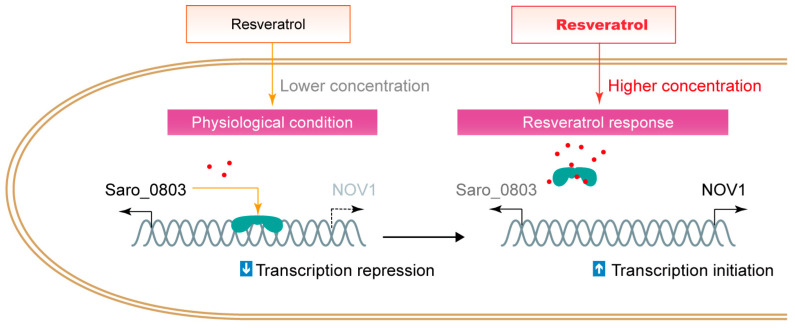
The schematic illustration of the interference of resveratrol to the repression of Saro_0803 to *nov1*.

Organisms, typically plants, produce molecules with antibiotic activities to encounter bacterial infection and damage, while certain bacteria co-evolve countermeasures to neutralize or consume the antibiotics. Particularly, Saro_0803 recognizes resveratrol and activates the NOV1 transcription to counteract high levels of resveratrol, which is different from certain MarR family regulators that employ a thiol group-mediated sensor mechanism to regulate bacterial stress responses [34,35,36,37]. It was also reported that Saro_0803, as a biosensor, could distinguish resveratrol from the precursors *p*-coumaric acid and trans-cinnamic acid as substrates and regulates the expression of the stilbene-degrading enzyme NOV1 [6] (Figure 6). By combining empirical observations, molecular docking, and mutagenesis-directed binding affinity assays, we propose that the aromatic-intensive core enclosed by residues F30, F43, F47, and W61 within Saro_0803 is a potential target for resveratrol to be trapped into. As this hydrophobic core is not directly engaged in DNA binding, a potential allosteric effect on Saro_0803 led by resveratrol binding is a possible reason for the resveratrol-induced inhibition of Saro_0803-DNA binding. More experimental evidence, nevertheless, will be needed to investigate these speculations. These results are an essential complement to the resveratrol-responsive mechanism of Saro_0803, which is the foundation of future modification of Saro_0803 as a biosensor with improved properties.

## Data Availability

The data that support the findings of this study are available from the corresponding author on reasonable request.

## Supplementary Materials

The following supporting information can be downloaded at: https://www.mdpi.com/article/10.3390/biom13030541/s1, Figure S1: Structural-based multisequence alignment of MarR family proteins featuring Saro_0803 from *Novosphingobium aromaticivorans* DSM 12444, MarR, EmrR, SlyA, HpaR; Figure S2: Comparison of the structural model of Saro_0803 with the crystal structure of MarR and SlyA; Figure S3: The sedimentation absorbance curve, residuals curve, and Sed coefficient curve detection by analytical ultracentrifugation experiments; Figure S4: One-dimensional [^1^H] spectrum of Saro_0803; Figure S5: Two-dimensional [^1^H-^15^N]-HSQC spectrum of Saro_0803; Figure S6: Two-dimensional [^1^H-^15^N]-HSQC spectrum of Saro_0803 with the increased concentration of ^15^N-labeled protein with 100, 250, 500 µM, and 1.0 mM; Figure S7: Binding assays via EMSA between Saro_0803 and four DNA fragments; Figure S8: Analytic size exclusion chromatography experiments to detect the protein–DNA interactions of Saro_0803 and its truncation Saro_0803^21-157^ to its operator DNA sequence *nov1p*^22bp^ utilizing Superdex 200 increase 10/300 GL column; Figure S9: EMSA analysis of the binding site of mutants of Saro_0803; Figure S10: The diagram of binding affinity (K_d_) and enthalpy values (ΔH) of mutants of Saro_0803 related to residues bound to the core DNA sequence *nov1p*^22bp^,detected by the performance of ITC experiments; ; Figure S11: EMSA analysis to investigate the ligand resveratrol perturbing the mutants of F30A, F43A, F47A, and W61A of Saro_0803 binding to the core sequence *nov1p*^22bp^. Figure S12: Chracterization of the binding capability of Saro_0803 mutants with *nov1p^22bp^*. Table S1: The PCR primers commercially synthesized for the plasmid constructs; Table S2: Oligonucleotides sequence commercially synthesized for double-strand DNA synthesis by annealing, truncation per 3 bp from the upstream of *nov1p*^58bp^; Table S3: Oligonucleotides sequence commercially synthesized for double-strand DNA synthesis by annealing, truncation per 3 bp from the downstream of *nov1p*^58bp^; Table S4: Oligonucleotides sequence commercially synthesized for double-strand DNA synthesis by annealing; truncation per 1 bp from the downstream of *nov1p*^25bp^; Table S5: ITC determination statistics of enthalpy term (ΔH), entropy term (−TΔS), Gibbs free energy (ΔG), and the stoichiometry N (sites).
