# Supplementary material for: Biophysical and Biochemical Characterization of the Binding of the MarR-like Transcriptional Regulator Saro_0803 to the nov1 Promotor and Its Inhibition by Resveratrol
Source: Biomolecules. 2023 Mar 16;13(3):541. doi: 10.3390/biom13030541 (PMC10046596; doi:10.3390/biom13030541)
Supplement: Supplementary file 1 [file biomolecules-13-00541-s001.zip › biomolecules-2218407-supplementary.pdf]

Supplementary Figures

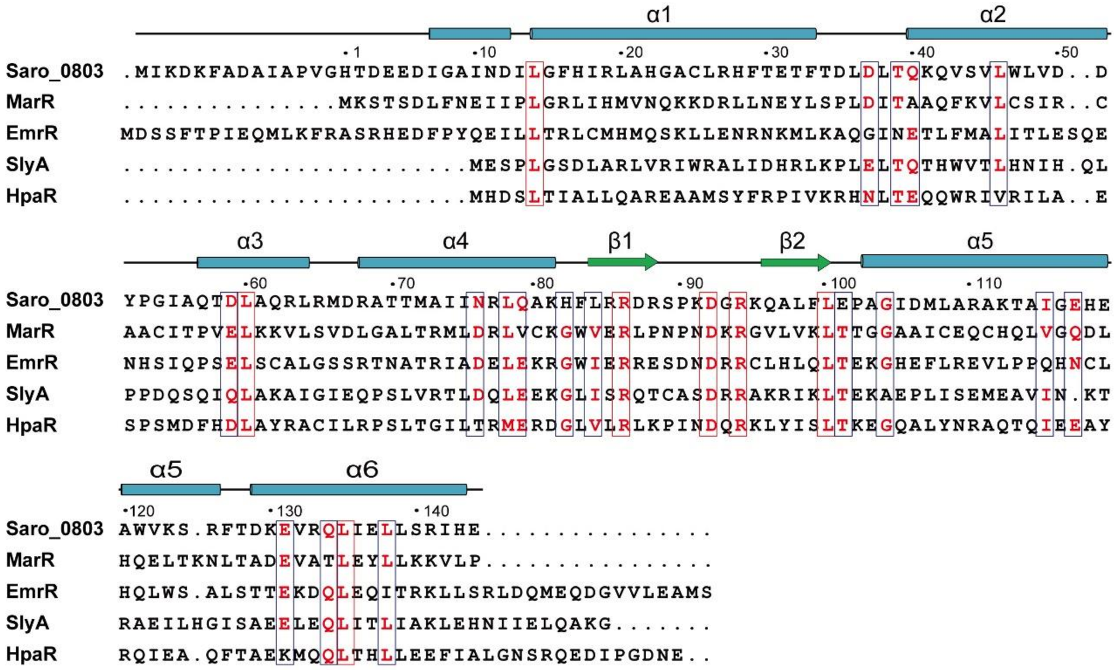

Figure. S1 Structural-based multisequence alignment of MarR family proteins featuring Saro_0803 from *Novosphingobium aromaticivorans* DSM 12444, MarR [1], EmrR [2], SlyA [3], HpaR (ID: WP_000543916.1). The conserved residues (indicated in red boxes) and the relatively conserved residues (indicated in blue boxes) [4].

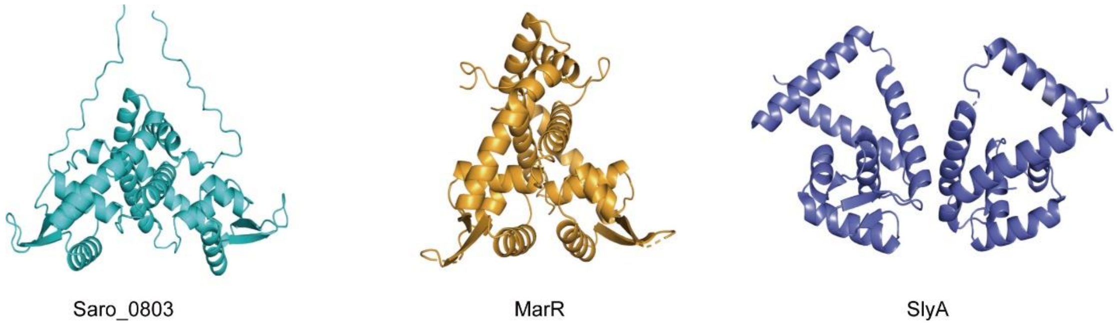

Figure. S2 Comparison of the structural model of Saro_0803 with the crystal structure of MarR [1] (PDB ID: 4JBA) and SlyA (PDB ID: 3DEU) [3], colored in cyan, orange and purple, respectively.

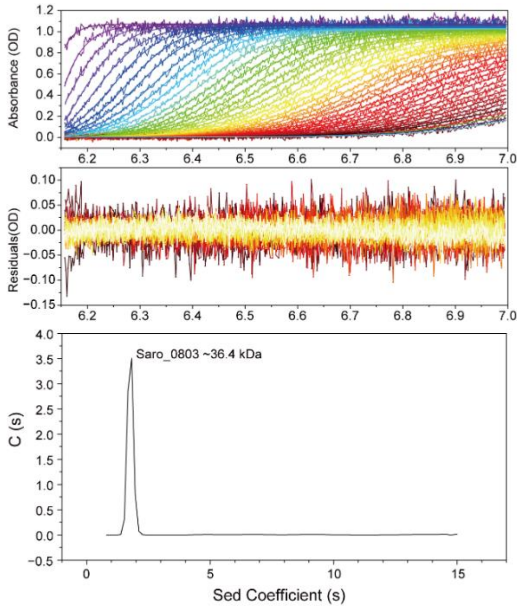

Figure. S3 The sedimentation absorbance curve (upper panel), residuals curve (OD, middle panel) and the Sed coefficient (s) curve (lower panel) detection by analytical ultracentrifugation (AUC) experiments.

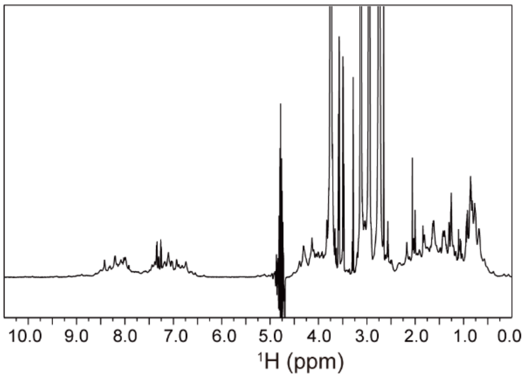

Figure. S4 One-dimensional [^{1}H] spectrum of Saro_0803.

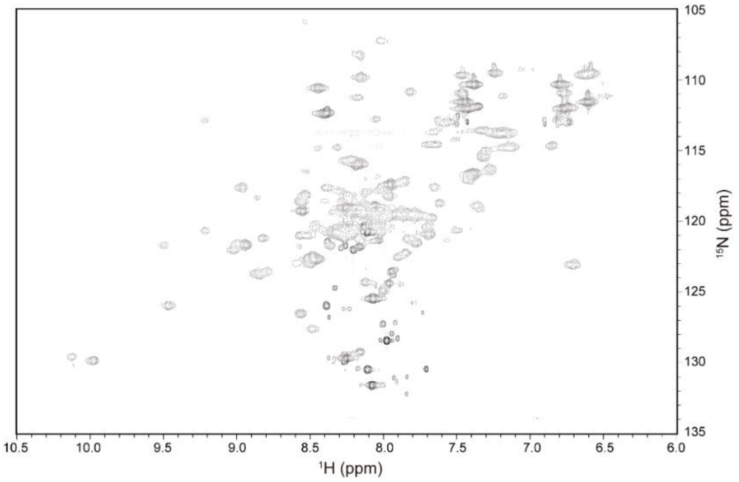

Figure. S5 Two-dimensional [^{1}H-^{15}N]-HSQC spectrum of Saro_0803.

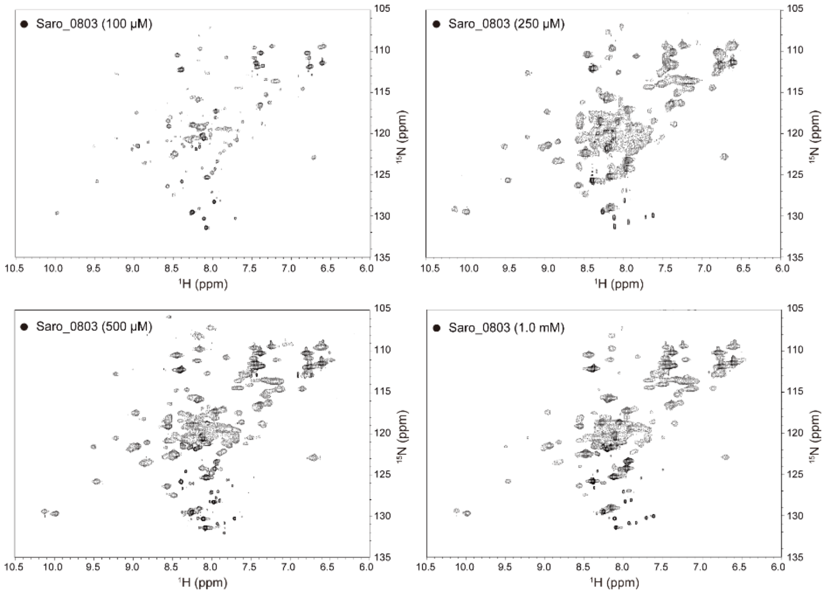

Figure. S6 2D [^{1}H-^{15}N]-HSQC spectra of Saro_0803 with the increased concentration of [^{15}N]-labeled protein with 100, 250, 500 μM and 1.0 mM.

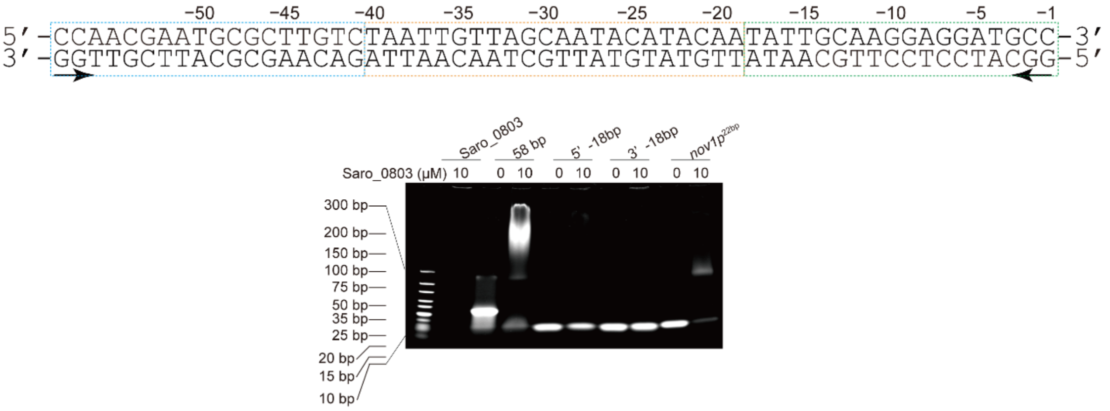

Figure. S7 Binding assays via EMSA between Saro_0803 and four DNA fragments: the 58-bp DNA fragment (box −58 to −1), *nov1p*^{22bp} (box −40 to −19), its 18-bp upstream DNA fragment (box −58 to −41), and its 18-bp downstream DNA fragment (box −18 to −1).

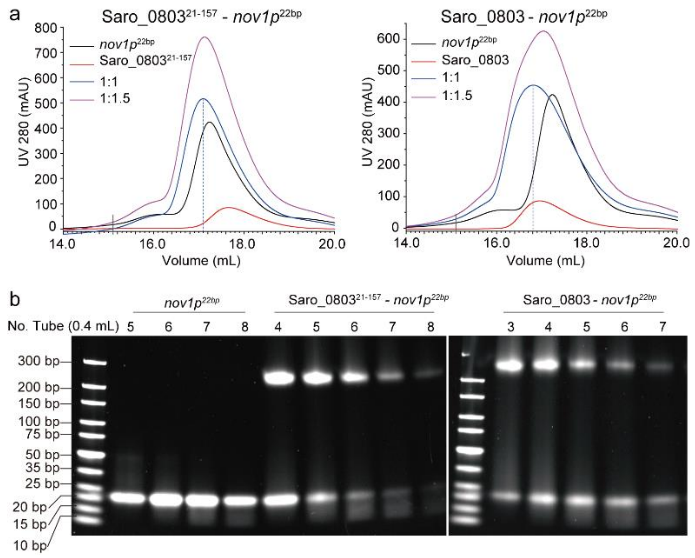

Figure. S8 Analytic size exclusion chromatography experiments to detect the protein–DNA interactions of Saro_0803 and its truncation Saro_0803$^{21\text{-}157}$ to its operator DNA sequence *nov1p*^{22bp} utilizing Superdex 200 increase 10/300 GL column (GE Healthcare, Chicago, IL, USA). The upper section (**a**) indicates the absorbance curve of DNA sequence *nov1p*^{22bp} with increased ratio to the absence and presence of Saro_0803 and its truncation Saro_0803$^{21\text{-}157}$. The lower section (**b**) indicates the EMSA analysis to confirm the migration of the binding curve at the ratio of 1:1, where the collection of elution begins at 15.1 mL with 0.4 mL per tube.

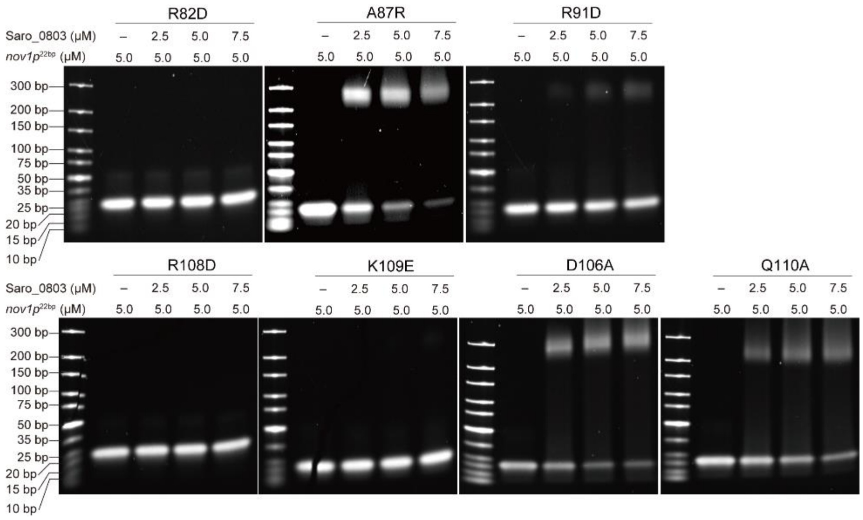

Figure. S9 EMSA analysis of the binding site of mutants of Saro_0803. The proteins with increased concentrations of 0, 2.5, 5.0, and 7.5 μM were incubated with 5.0 μM DNA sequence *nov1p*^{22bp}.

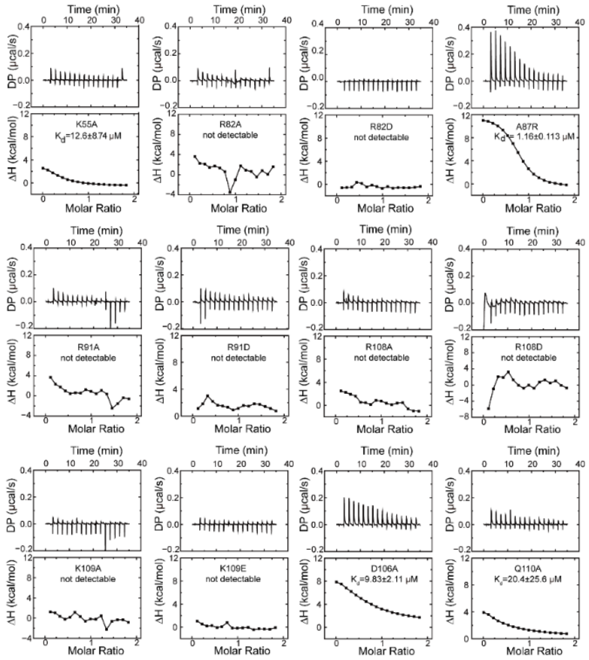

Figure. S10 The diagram of binding affinity (K_d) and enthalpy values (ΔH) of mutants of Saro_0803, related to residues bound to the core DNA sequence *nov1p*^{22bp}, detected by the performance of ITC experiments.

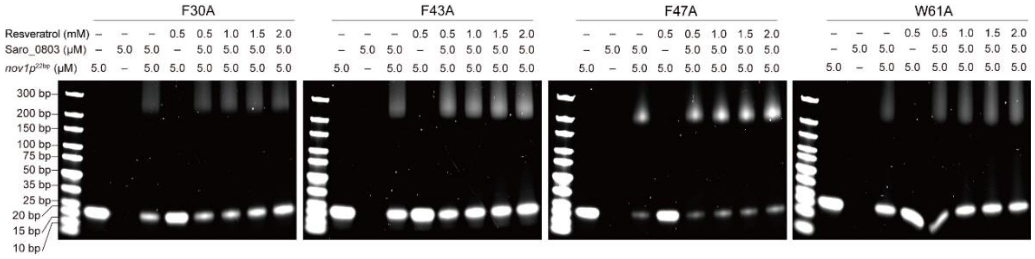

Figure. S11 EMSA analysis to investigate the ligand resveratrol perturbing the mutants of F30A, F43A, F47A and W61A of Saro_0803 binding to the core sequence *nov1p*^{22bp}. Incrementally increased concentrations of resveratrol (0, 0.5, 1.0, 1.5, 2.0 mM) were incubated with 5.0 μM mutated protein-*nov1p*^{22bp} complex.

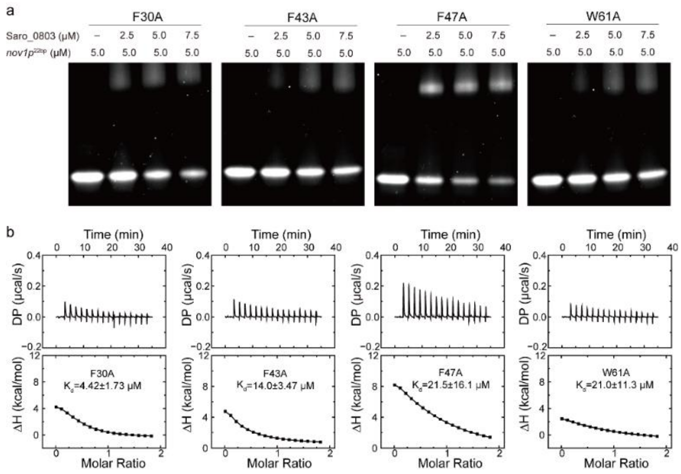

Figure. S12 Chracterization of the binding capability of Saro_0803 mutants with *nov1p*^{22bp}. **(a)** EMSA analysis of the mutants related to the hydrophobic cavity of Saro_0803 to *nov1p*^{22bp} . **(b)** ITC assay with continuous titration of *nov1p*^{22bp} into Saro_0803 mutants. The proteins with increased concentrations of 0, 2.5, 5.0, and 7.5 μM were incubated with 5.0 μM DNA sequence *nov1p*^{22bp} and the diagram of binding affinity (K_d) and enthalpy values (ΔH) of mutants of Saro_0803, related to residues bound to the core DNA sequence *nov1p*^{22bp}, detected by the performance of ITC experiments.

Supplementary Tables

Table. S1 The PCR primers commercially synthesized for the plasmid constructs.

Name (vector pET 30a+)	Sequence(5'-3')
Saro_0803-F	GGAATTCCATATGATCAAAGATAAATTTGCGGACGC
Saro_0803-R	CCGCTCGAG TTCGTGAATGCGAGACAGC
Saro_0803^{F30A}-F	GATCAACGATATTCTGGGTGCCCATATTCGTCTGGCCCA
Saro_0803^{F30A}-R	TGGGCCAGACGAATATGGGCACCCAGAATATCGTTGATC
Saro_0803^{F43A}-F	GCATGCCTGCGTCACGCTACCGAAACGTTCACCGA
Saro_0803^{F43A}-R	TCGGTGAACGTTTCGGTAGCGTGACGCAGGCATGC
Saro_0803F47A-F	CGTCACTTTACCGAAACGGCCACCGATCTGGACCTGAC
Saro_0803^{F47A}-R	GTCAGGTCCAGATCGGTGGCCGTTTCGGTAAAGTGACG
Saro_0803^{K55A}-F	TCTGGACCTGACGCAGGCACAAGTGAGTGTTCTGTGG
Saro_0803^{K55A}-R	CCACAGAACACTCACTTGTGCCTGCGTCAGGTCCAGA
Saro_0803^{W61A}-F	AGAAACAAGTGAGTGTTCTGGCGCTGGTCGATGACTATCC
Saro_0803^{W61A}-R	GGATAGTCATCGACCAGCGCCAGAACACTCACTTGTTTCT
Saro_0803^{R82A}-F	GTCTGCGCATGGACGCTGCTACCACGATGG
Saro_0803^{R82A}-R	CCATCGTGGTAGCAGCGTCCATGCGCAGAC
Saro_0803^{R82D}-F	AACGTCTGCGCATGGACGATGCTACCACGATGGCGA
Saro_0803^{R82D}-R	TCGCCATCGTGGTAGCATCGTCCATGCGCAGACGTT
Saro_0803^{A87R}-F	CCGTGCTACCACGATGCGGATTATCAATCGCCTGCAAG
Saro_0803^{A87R}-R	CTTGCAGGCGATTGATAATCCG CATCGTGGTAGCACGG
Saro_0803^{R91A}-F	CGATGGCGATTATCAATGCCCTGCAAGCCAAAC
Saro_0803^{R91A}-R	GTTTGGCTTGCAGGGCATTGATAATCGCCATCG
Saro_0803^{R91D}-F	CACGATGGCGATTATCAATGACCTGCAAGCCAAACATTTTCT
Saro_0803^{R91D}-R	AGAAAATGTTTGGCTTGCAGGTCATTGATAATCGCCATCGTG
Saro_0803^{D106A}-F	CGATCGTAGCCCGAAAGCCGGCCGCAAACAAGCTC
Saro_0803^{D106A}-R	GAGCTTGTTTGCGGCCGGCTTTCGGGCTACGATCG
Saro_0803^{R108A}-F	AGCCCGAAAGACGGCGCCAAACAAGCTCTGTTCCTGG
Saro_0803^{R108A}-R	CCAGGAACAGAGCTTGTTTGGCGCCGTCTTTCGGGCT
Saro_0803^{R108D}-F	TAGCCCGAAAGACGGCGACAAACAAGCTCTGTTCCTGG
Saro_0803^{R108D}-R	CCAGGAACAGAGCTTGTTTGTCGCCGTCTTTCGGGCTA
Saro_0803^{K109A}-F	CGAAAGACGGCCGCGCACAAGCTCTGTTCCTGG
Saro_0803^{K109A}-R	CCAGGAACAGAGCTTGTGCGCGGCCGTCTTTCG
Saro_0803^{K109E}-F	GAAAGACGGCCGCGAACAAGCTCTGTTC
Saro_0803^{K109E}-R	GAACAGAGCTTGTTCGCGGCCGTCTTTC
Saro_0803^{Q110A}-F	GAAAGACGGCCGCAAAGCAGCTCTGTTCCTGGAAC
Saro_0803^{Q110A}-R	GTTCCAGGAACAGAGCTGCTTTGCGGCCGTCTTTC

Table. S2 The Oligonucleotides sequence commercially synthesized for double-strand DNA synthesis by annealing, with truncations presented per 3 bp from the upstream of *nov1p*^{58bp}.

Name	DNA sequence(5'-3')
nov1p^{58bp}	CCAACGAATGCGCTTGTCTAATTGTTAGCAATACATACAATATTGCAAGGAGGATGCC
	GGCATCCTCCTTGCAATATTGTATGTATTGCTAACAATTAGACAAGCGCATTCGTTGG
**Truncation per 3bp from the upstream of *nov1p*^{58bp}**	
5'-Forward: −3bp	ACGAATGCGCTTGTCTAATTGTTAGCAATACATACAATATTGCAAGGAGGATGCC
	GGCATCCTCCTTGCAATATTGTATGTATTGCTAACAATTAGACAAGCGCATTCGT
5'-Forward: −6bp	AATGCGCTTGTCTAATTGTTAGCAATACATACAATATTGCAAGGAGGATGCC
	GGCATCCTCCTTGCAATATTGTATGTATTGCTAACAATTAGACAAGCGCATT
5'-Forward: −9bp	GCGCTTGTCTAATTGTTAGCAATACATACAATATTGCAAGGAGGATGCC
	GGCATCCTCCTTGCAATATTGTATGTATTGCTAACAATTAGACAAGCGC
5'-Forward: −12bp	CTTGTCTAATTGTTAGCAATACATACAATATTGCAAGGAGGATGCC
	GGCATCCTCCTTGCAATATTGTATGTATTGCTAACAATTAGACAAG
5'-Forward: −15bp	GTCTAATTGTTAGCAATACATACAATATTGCAAGGAGGATGCC
	GGCATCCTCCTTGCAATATTGTATGTATTGCTAACAATTAGAC
5'-Forward: −18bp	TAATTGTTAGCAATACATACAATATTGCAAGGAGGATGCC
	GGCATCCTCCTTGCAATATTGTATGTATTGCTAACAATTA
5'-Forward: −21bp	TTGTTAGCAATACATACAATATTGCAAGGAGGATGCC
	GGCATCCTCCTTGCAATATTGTATGTATTGCTAACAA
5'-Forward: −24bp	TTAGCAATACATACAATATTGCAAGGAGGATGCC
	GGCATCCTCCTTGCAATATTGTATGTATTGCTAA
5'-Forward: −27bp	GCAATACATACAATATTGCAAGGAGGATGCC
	GGCATCCTCCTTGCAATATTGTATGTATTGC
5'-Forward: −30bp	ATACATACAATATTGCAAGGAGGATGCC
	GGCATCCTCCTTGCAATATTGTATGTAT
5'-Forward: −33bp	CATACAATATTGCAAGGAGGATGCC
	GGCATCCTCCTTGCAATATTGTATG
5'-Forward: −36bp	ACAATATTGCAAGGAGGATGCC
	GGCATCCTCCTTGCAATATTGT

Table. S3 The Oligonucleotides sequence commercially synthesized for double-strand DNA synthesis by annealing, with truncations presented per 3 bp from the downstream of *nov1p*^{58bp}.

Name	DNA sequence(5'-3')
nov1p^{58bp}	CCAACGAATGCGCTTGTCTAATTGTTAGCAATACATACAATATTGCAAGGAGGATGCC
	GGCATCCTCCTTGCAATATTGTATGTATTGCTAACAATTAGACAAGCGCATTCGTTGG
**Truncation per 3bp from the downstream of *nov1p*^{58bp}**	
3'-Reverse: −3bp	CCAACGAATGCGCTTGTCTAATTGTTAGCAATACATACAATATTGCAAGGAGGAT
	ATCCTCCTTGCAATATTGTATGTATTGCTAACAATTAGACAAGCGCATTCGTTGG
3'-Reverse: −6bp	CCAACGAATGCGCTTGTCTAATTGTTAGCAATACATACAATATTGCAAGGAG
	CTCCTTGCAATATTGTATGTATTGCTAACAATTAGACAAGCGCATTCGTTGG
3'-Reverse: −9bp	CCAACGAATGCGCTTGTCTAATTGTTAGCAATACATACAATATTGCAAG
	CTTGCAATATTGTATGTATTGCTAACAATTAGACAAGCGCATTCGTTGG
3'-Reverse: −12bp	CCAACGAATGCGCTTGTCTAATTGTTAGCAATACATACAATATTGC
	GCAATATTGTATGTATTGCTAACAATTAGACAAGCGCATTCGTTGG
3'-Reverse: −15bp	CCAACGAATGCGCTTGTCTAATTGTTAGCAATACATACAATAT
	ATATTGTATGTATTGCTAACAATTAGACAAGCGCATTCGTTGG

Table. S4 The Oligonucleotides sequence commercially synthesized for double-strand DNA synthesis by annealing, with truncations presented per 1 bp from the downstream of *nov1p*^{25bp}.

Name	DNA sequence(5'-3')
nov1p^{25bp}	TAATTGTTAGCAATACATACAATAT
	ATATTGTATGTATTGCTAACAATTA
**Truncation per 1bp from the downstream of *nov1p*^{25bp}**	
3'-Reverse: −1bp	TAATTGTTAGCAATACATACAATA
	TATTGTATGTATTGCTAACAATTA
3'-Reverse: −2bp	TAATTGTTAGCAATACATACAAT
	ATTGTATGTATTGCTAACAATTA
3'-Reverse: −3bp	TAATTGTTAGCAATACATACAA
	TTGTATGTATTGCTAACAATTA
3'-Reverse: −4bp	TAATTGTTAGCAATACATACA
	TGTATGTATTGCTAACAATTA

Table. S5 The ITC determination statistics of enthalpy term (ΔH), entropy term (−TΔS), Gibbs free energy (ΔG) and the stoichiometry N (sites).

the ITC determination					
	Kd(M)	△H (kcal/mol)	−T△S(kcal/mol)	△G (kcal/mol)	N (sites)
Ctrl	NA	NA	NA	NA	NA
Saro_0803	3.57e−6±703e−9	6.70±0.463	−14.1	-7.43	0.923±3.3e−2
F30A	4.42e−6±1.73e−6	6.98±1.48	−14.3	−7.31	0.439±4.2e−2
F43A	14.0e−6±3.47e−6	37.2±44.3	−43.9	−6.62	0.101±9.4e−2
F47A	21.5e−6±16.1e−6	22.7±15.1	−29	−6.37	0.720±0.138
K55A	12.2e−6±8.97e−6	12.2±15.7	−18.9	−6.7	0.339±4.8e−2
W61A	21.0e−6±11.3e−6	12.5±11.7	−18.9	−6.38	0.144±2.24
R82A	NA	NA	NA	NA	NA
R82D	NA	NA	NA	NA	NA
A87R	1.16e−6±113e−9	12.5±0.275	−20.6	−8.1	0.771±8.8e−3
R91A	NA	NA	NA	NA	NA
R91D	NA	NA	NA	NA	NA
R108A	45.6e−6±166e−6	11.7±47.0	−17.6	−5.92	0.947±1.18
R108D	NA	NA	NA	NA	NA
K109A	NA	NA	NA	NA	NA
K109E	NA	NA	NA	NA	NA
D106A	9.83e−6±2.11e−6	12.8±1.75	−19.6	−6.83	0.640±2.5e−2
Q110A	20.4e−6±25.6e−6	15.1±33.3	−21.5	−6.4	0.134±1.94
notes: the binding determination of Saro_0803 and related mutants to the *nov1p*^{22bp}					

the ITC determination					
	Kd(M)	△H (kcal/mol)	−T△S(kcal/mol)	△G (kcal/mol)	N (sites)
Ctrl	NA	NA	NA	NA	NA
F30A	NA	NA	NA	NA	NA
F43A	NA	NA	NA	NA	NA
F47A	NA	NA	NA	NA	NA
W61A	278e−6±256e−6	−0.247±0.179	−4.6	−4.85	10.0±1.09
Saro_0803	35.7e−6±7.71e−6	−0.235±1.9e−2	−5.83	−6.07	7.15±0.235
notes: the binding determination of Saro_0803 and related mutants to the resveratrol					

Reference

1 Hao, Z. *et al.* The multiple antibiotic resistance regulator MarR is a copper sensor in Escherichia coli. *Nat Chem Biol* 2014, **10**, 21-28. https://doi.org:10.1038/nchembio.1380

2 Lomovskaya, O.; Lewis, K.; Matin, A. EmrR is a negative regulator of the Escherichia coli multidrug resistance pump EmrAB. *J Bacteriol* 1995, **177**, 2328-2334. https://doi.org:10.1128/jb.177.9.2328-2334.1995

3 Will, W. R. *et al.* The Evolution of SlyA/RovA Transcription Factors from Repressors to Countersilencers in Enterobacteriaceae. *mBio* 2019, **10**. https://doi.org:10.1128/mBio.00009-19

4 Altschul, S. F. *et al.* Gapped BLAST and PSI-BLAST: a new generation of protein database search programs. *Nucleic Acids Res* 1997, **25**, 3389-3402. https://doi.org:10.1093/nar/25.17.3389
